# Perspectives on Iron Oxide-Based Materials with Carbon as Anodes for Li- and K-Ion Batteries

**DOI:** 10.3390/nano12091436

**Published:** 2022-04-22

**Authors:** Mario Valvo, Christina Floraki, Elie Paillard, Kristina Edström, Dimitra Vernardou

**Affiliations:** 1 Ångström Laboratory, Department of Chemistry, Uppsala University, SE-751 21 Uppsala, Sweden; kristina.edstrom@kemi.uu.se; 2 Department of Electrical and Computer Engineering, School of Engineering, Hellenic Mediterranean University, 71410 Heraklion, Greece; mtk25@edu.hmu.gr; 3 Politecnico di Milano, Department of Energy, Via Lambruschini 4, 20156 Milan, Italy; elieelisee.paillard@polimi.it; 4 Institute of Emerging Technologies, Hellenic Mediterranean University, 71410 Heraklion, Greece

**Keywords:** anode, iron oxide, lithium-ion battery, potassium-ion battery, non-aqueous electrolyte

## Abstract

The necessity for large scale and sustainable energy storage systems is increasing. Lithium-ion batteries have been extensively utilized over the past decades for a range of applications including electronic devices and electric vehicles due to their distinguishing characteristics. Nevertheless, their massive deployment can be questionable due to use of critical materials as well as limited lithium resources and growing costs of extraction. One of the emerging alternative candidates is potassium-ion battery technology due to potassium’s extensive reserves along with its physical and chemical properties similar to lithium. The challenge to develop anode materials with good rate capability, stability and high safety yet remains. Iron oxides are potentially promising anodes for both battery systems due to their high theoretical capacity, low cost and abundant reserves, which aligns with the targets of large-scale application and limited environmental footprint. However, they present relevant limitations such as low electronic conductivity, significant volume changes and inadequate energy efficiency. In this review, we discuss some recent design strategies of iron oxide-based materials for both electrochemical systems and highlight the relationships of their structure performance in nanostructured anodes. Finally, we outline challenges and opportunities for these materials for possible development of KIBs as a complementary technology to LIBs.

## 1. Introduction

Lithium-ion batteries (LIBs) have gained tremendous momentum in the energy storage market due to their substantial advantages including low self-discharge, high stability, high energy density reaching about 250 Wh kg^−1^ at the cell level (18650-type cells) and low cost of 200 € kW h^−1^ [1,2,3]. Nevertheless, an increasing need for energy storage has directed studies towards the development of materials with improved cycling stability, higher capacity values and charge/discharge rates [4,5,6]. Despite these hopeful perspectives, some limitations including uneven geographical distribution of lithium and criticality of some other LIB components such as cobalt exist as well [3]. Creating alternative energy storage systems with complementary functionalities is therefore essential. Potassium ion batteries (KIBs) have attracted noticeable interest due to their potential low cost, great abundance and similarities of physical and chemical properties of K with those of Li [7]. Potassium possesses a suitable potential vs. the standard hydrogen electrode (SHE) of −2.92 V compared to Li (−3.04 V vs. SHE) [8]. However, a bigger ionic radius for K (0.138 nm vs. 0.076 nm for Li) results in a lower energy and power density [9]. Hence, exploring anode materials that could give high capacity, good lifespan, enhanced capability and rate performance is a great challenge. Metal oxides have been proposed as very attracting candidates since they could deliver reversible capacities two to three times higher than that of conventional graphite in LIBs [10]. Nevertheless, metal oxides display a different type of reaction with Li^+^ ions compared to that of graphite that relies on a topotactic mechanism of ion insertion preserving its lattice from major structural variations. Metal oxides instead undergo so-called reconstitution reactions upon lithiation, in which the metal–oxygen bond is broken (i.e., conversion) and their elemental components are irreversibly displaced with possible concurrent amorphization of the reaction products. The latter typically consist of Li_2_O and a nanoparticulated form of the displaced metal originally present in the pristine metal oxide.

Depending on the nature of the displaced metal particles, two distinctive reactions are possible, i.e., subsequent Li-alloying of the metal [11] or sheer formation/decomposition of Li_2_O associated with the reduction and oxidation of the metal nanoparticles [12]. A typical advantage of metal oxides such as, for example, SnO_2_ or Sb_2_O_3_ is their very high capacity due to an enhanced lithium storage derived from both their initial conversion and further Li-alloying. However, an intrinsic disadvantage is their poor reversibility in the first cycle due to their conversion and irreversible formation of Li_2_O, which becomes practically inactive. This has represented one of the main obstacles in attempting to substitute graphite with these alternative anode materials with a much higher capacity, due to such remarkable Coulombic losses. Another severe limitation for this type of combined Li-alloying metal oxide is their large volume expansion/contraction (e.g., >100%) upon lithium uptake/release, which drastically undermines their mechanical stability upon repeated cycling and often results in premature electrode failure. To some extent, Li_2_O can act as a surrounding ‘buffering matrix’ [11] for the Li-alloying/de-alloying nanoparticles, yet their cumulative volume variations remain challenging for maintaining electrode integrity upon long-term cycling.

On the other hand, most transition metal oxides do not undergo Li-alloying after their initial conversion and their volume variations are more contained, offering a unique possibility of using Li_2_O as an active medium for Li storage, thanks to its mostly reversible behavior linked to a ‘catalytic effect’ of these small transition metal nanoparticles. The latter assist Li_2_O decomposition and ease the release of Li^+^ ions upon subsequent oxidation. Nevertheless, the main disadvantage of transition metal oxides remains a pronounced voltage hysteresis between their lithiation/de-lithiation, which results in a scarce energy efficiency in each cycle and occurs over a wide range of potentials. These detrimental aspects, together with a remarkable initial formation of a surface passivation layer have impeded their use as potential alternative anodes to graphite in LIBs.

Among transition metal oxides, iron oxides are particularly appealing, as they are plentiful, eco-friendly, non-toxic and low cost. Iron is an earth-abundant element that can be found almost everywhere in the world, with iron derivatives having the lowest prices compared with others [13]. Taking this as an advantage, iron oxides such as Fe_2_O_3_ and Fe_3_O_4_ have been investigated in LIBs due to their respective high specific capacities (i.e., 1007 mAh g^−1^ and 926 mAh g^−1^, assuming a theoretical uptake of 6 Li and 8 Li per formula unit, respectively) compared with that of graphite (372 mAh g^−1^) [14,15]. Despite this, one of the most challenging issues for Fe oxides is to maintain their electrochemical stability during prolonged cycling. Therefore, several investigations have focused on various synthesis procedures in order to control their particle size, shape, agglomeration and overall electrode structure to ensure improved mechanical and electrical properties during the electrochemical reactions with the goal of enhancing their performance and durability upon repeated charge/discharge cycles. Iron oxides have been broadly examined for potential use in advanced LIBs, while their study in KIBs is still at the beginning [16]. Independently of the lithium or potassium cell chemistry, a series of challenges for iron oxides needs to be overcome, including low ionic conductivity, limited Coulombic efficiency due to unstable solid electrolyte interphase (SEI) [17], high polarization between charge/discharge in each cycle, which is crucial for the energy efficiency of the battery [15], and large volume variations during conversion/de-conversion processes [18], in line with the above points.

Various strategies have been employed to deal with the abovementioned challenges. A synergistic effect of two materials can typically provide better physicochemical properties, high stability and storage performance than single ones [19]. For instance, an anode material based on nanoparticulated Fe_2_O_3_ and Co_3_O_4_ provided a capacity of 220 mAh g^−1^ for KIB, yet with poor intrinsic conductivity and severe volume expansion/contraction during the K^+^ uptake/removal processes [16]. Indeed, an approach relying on nanostructured anode materials for these compounds comes along with significant advantages including a quicker mass transport of ions and convenient relief of internal stress [20]. Nanostructuring eases the motion of the charge carriers thanks to reduced path lengths in nano-sized active materials, simultaneously boosting their electrochemical reactivity due to an increased surface-to-volume ratio for their constitutive atoms. Even so, a neat improvement of the electronic conductivity is still important for high power density batteries [15] and this typically represents a limiting factor for iron oxides. For instance, Fe_2_O_3_ is a poor electron conductor due to its semiconductor nature (E_gap_ ≈ 1.9 - 2.2 eV), while Fe_3_O_4_ displays only a moderate electrical conductivity (σ_el_ ≈ 2 × 10^2^ S cm^−1^). However, dramatic structural and compositional changes upon conversion/de-conversion unavoidably affect this crucial aspect of electronic conductivity. In order to deal with this issue, a combination of iron oxides with carbon coatings has been shown to improve the structural stability and, consequently, the electrochemical performance of iron oxide active particles, in which the conversion/de-conversion occurs within the volume enclosed by the carbon shell. This has a three-fold advantage: (i) It directly provides suitable electronic wiring at the active particle level during the reactions; (ii) it helps contain the volume variations, as the carbon coating often encapsulates the iron oxide particles; and (iii) it simultaneously acts as an effective barrier against particle agglomeration upon prolonged cycling. It was found, for example, that Fe_3_O_4_/rGO presents a sufficient cycling stability and rate capability (917.4 mAh g^−1^ with ~52% capacity retention at 1.0 A g^−1^) in Li half-cells due to an ultrafine hollow structure of Fe_3_O_4_ and the presence of conductive reduced graphene oxide (rGO) shortening the ion diffusion paths and favoring the electron transfer [15]. Extraordinary behavior was also observed for Fe_2_O_3_/N-rGO in LIBs with a reversible capacity of 1398 mAh g^−1^ at 0.5 A g^−1^ after 200 cycles. This performance is due to the N-rGO sheets that could effectively act as a buffer layer to avoid structural failure during the ion uptake/removal processes [21].

One may then realize that a plethora of studies is currently available regarding the suitability of iron oxide-based anodes for LIBs, yet there is still little information for these materials with respect to possible similar applications for KIBs. In this review paper, studies of iron oxide-based materials published in recent years for LIBs and KIBs are presented, including synthesis design routes to obtain anodes with enhanced performances. Additionally, challenges and opportunities for these compounds are outlined for a potential future development of KIBs as a complementary technology to LIBs.

## 2. Basic Requirements of Electrode Materials

While battery technology has met significant advancements in performance, there are still matters to handle for their widespread application from portable electronic devices to electric vehicles. One of the challenges considers the development of electrode materials with the following requirements [22,23,24]:

➢ High gravimetric and volumetric capacities, although these features may not be critical for stationary storage of electricity.
➢ Adequate electronic and ionic conductivities to support the electrochemical reactions and grant access to good rate capabilities.
➢ Short diffusion lengths for the charge carriers to provide an enhanced charge transfer via a higher number of surface active sites in nanostructures, thus yielding shorter diffusion times for both electrons and ions to conveniently sustain high charge–discharge rates.
➢ A low anode redox potential in combination with a high-potential cathode to provide high cell voltage. The anodic redox potential should not be too low to avoid incidental uneven metal plating, which detrimentally affects both safety (risk of internal short circuits) and performances (electrolyte consumption and power loss) of the cell.
➢ High mechanical strength of the active materials and composite electrode coatings, especially when considerable volume variations are involved in the reactions.
➢ High reversibility of the electrochemical reactions to maintain their specific charge and energy for hundreds of cycles in order to ensure cell durability and adequate power delivery.
➢ Stability of the solid electrolyte interphase (SEI) on the anode to limit side reactions and ensure high Coulombic efficiency, preventing simultaneous rise of cell internal resistance with consequent loss of power.
Elevated thermal stability to support a wide window of operation temperatures and prevent earlier electrolyte degradation at the reacting electrode interfaces.
➢ Abundant, non-toxic and environmentally friendly materials that can match with cost-effective electrode coating procedures.
➢ Increased safety and optimized cell design to facilitate materials recovery/re-use at the time of battery disposal.
➢ Low cost and efficient fabrication of both active materials and electrodes to enable potential large-scale use of corresponding cells.

In the following sections, the basic principles of LIBs and KIBs are briefly shown along with some recent synthetic strategies of iron oxides for both systems. Special attention will be paid to electrode modifications to enhance their stability and ion mobility.

## 3. Li-Ion Batteries

Generally, a battery contains one or more electrochemical cells connected in parallel (to increase current), in series (to increase voltage) or in combined configurations, and transforms chemical energy into electrical energy. In the case of a rechargeable LIB, the latter is composed of a cathode, an anode and an electrolyte containing Li^+^ with a thin layer of insulating material—called the separator—in between them. The separator allows the Li^+^ to pass through, while it blocks the electrons, averting the contact between the two electrodes, which otherwise would result in a short-circuit. Both cathode and anode materials are typically employed as a composite coated layer onto an Al and a Cu current collector, respectively. The cell potential is determined by the difference between the chemical potential of the Li in the anode and the cathode. Hence, an appropriate selection of both active materials for cathodic and anodic purposes, as well as a compatibility at the electrode–electrolyte level are essential to increase the energy, power density and life cycle of the battery.

**Figure 1:**
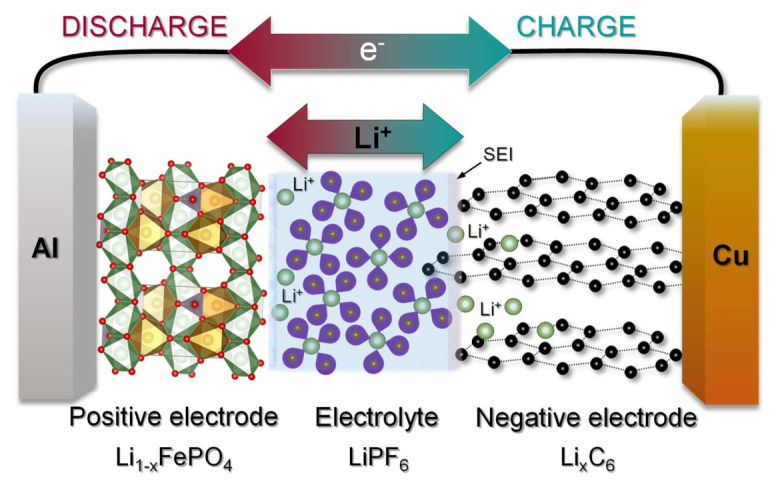
A schematic representation of the basic principles of operation of rechargeable LIBs utilizing lithium hexafluorophosphate (LiPF_6_) as conventional electrolytic salt. The latter is typically dissolved in organic-based solvent mixtures (e.g., ethylene carbonate/diethyl carbonate) representing a class of electrolytic solutions which play a key role in the process of suitable SEI layer formation on the negative electrode. Note that also other types of electrolytic salts and organic solvents could be employed to obtain electrolytic solutions with different physiochemical properties.

Figure 1 shows the basic principle of operation of a Li^+^ battery cell. The basic design of Li-ion cells is essentially the same as those commercialized two decades ago by Sony with a continuous and extensive exploration of electrode materials, electrolytes and separators [25]. During charging, Li^+^ are directed from the cathode through the electrolyte towards the anode [25]. Meanwhile, the electrons travel from the cathode to the anode through an external electric circuit in order to maintain charge neutrality. The potential of Li is much higher in the anode than in the cathode and consequently the electrical energy is stored in the form of chemical energy. The process is inverted once the battery is discharged, with respective reversed fluxes of Li^+^ in the electrolyte and e^−^ in the external circuit, through which the electrochemical energy is released in the form of electrical energy. The success, unrivalled performances and high reversibility of current LIBs are mainly due to their elegant use of Li insertion reactions, which do not involve major structural re-arrangements and provoke only contained volume changes (i.e., <10%) during the charge/discharge processes, as well as an ability to form and consolidate a protective passivation layer (SEI) on the graphite electrode in carbonate-based organic electrolytes. The SEI layer effectively prevents a detrimental process of continuous electrolyte reduction and its irreversible consumption due to an inherent instability of the electrolyte in the range of low operation potentials vs. Li^+^/Li. This protective action is accomplished through its electronically insulating nature that provides a barrier against electrons potentially triggering further electrolyte reduction and its ion-conduction properties that simultaneously enable suitable movements of the Li^+^ ions during their incorporation/release into/from the electrode material.

## 4. K-Ion Batteries

**Figure 2:**
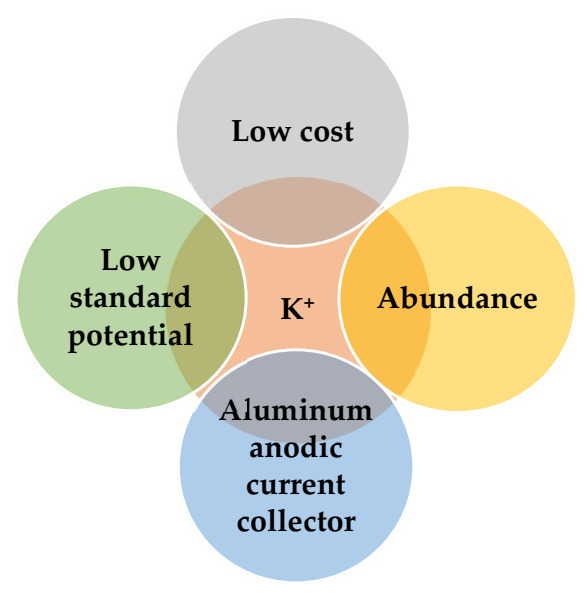
Some advantages of K^+^ for their implementation in rechargeable K-ion batteries.

KIBs have attracted increasing attention due to the availability of abundant potassium resources comprising ~2.09% of the earth’s crust by weight [26]. This can ensure satisfactory supply and low cost of raw materials (Figure 2) for their possible future fabrication. Another reason for the development of KIBs is their higher voltage (i.e., up to 0.2–0.3 V) compared with that of sodium-ion batteries (NIBs) [8]. In addition, it was proved that the standard potential of K^+^/K is slightly more negative (−3.002 V) than that of Li^+^/Li (−2.906 V) in propylene carbonate (PC) as solvent [27]. This might be possibly one advantage of K over Li in non-aqueous systems, thus potentially bringing a wider voltage window and, hypothetically, a higher energy density than LIBs, assuming that the operating potential of the cathode is analogous to that of LIBs. However, it should be remembered that the specific capacities (i.e., gravimetric and volumetric) of both cathode and anode should also be taken into account, as well as the fact that the atomic mass of potassium is much higher than that of lithium. Hence, both aspects of operation voltage and effective capacity need to be thoroughly considered to judge accurately the ultimate energy density content of these cells. KIBs are also expected to possibly support high power densities based on elevated diffusion rates of K^+^ ions which typically possess rather weak Coulombic interactions [28].

The use of K^+^ can also enable Al as a cost-effective anodic current collector replacing Cu, since K does not alloy with Al, contrary to Li, thus requiring only one type of metal for this purpose and easing possible cell component recycling. This is an important advantage that KIBs share with NIBs [28,29] and that could further contribute to making these rechargeable complementary technologies possibly competitive in terms of limited costs for their raw materials and contained environmental footprint in a future perspective of potential development of their industrial chains and manufacturing.

The basic principle of operation of KIBs is similar to the one depicted above for LIBs and it is based on the reversible movement of K^+^ between the anode and cathode materials accompanied by the simultaneous motion of the electrons in the outer circuit [30].

Nevertheless, KIB technology is still in an embryo stage and the challenges for its future development appear rather tough from a series of different perspectives. While a use of similar types of materials, which have broadly been investigated for LIBs, can represent an advantage, particularly from the point of view of possible utilization of graphite as anodic insertion host for K^+^, in practice a number of limitations exist for these cells and especially for their negative electrode side and its reactive interface with the electrolyte. Hence, it can be anticipated that the quest for alternative types of anodes for KIBs relying on different materials than graphite carries along all its previous issues and opens up additional challenges due to their diverse reaction mechanisms.

## 5. Iron Oxides for LIBs and KIBs

### 5.1. Li Insertion and Conversion of Iron Oxides

Among several types of iron oxides and oxyhydroxides that have extensively been investigated for possible use in LIBs, hematite (α-Fe_2_O_3_) [31] and magnetite (Fe_3_O_4_) have typically attracted significant attention due to high theoretical specific capacity and moderate electronic conductivity, respectively. Hematite possesses a corundum-type crystal structure with iron in its Fe(III) oxidation state, while magnetite displays an inverse spinel structure in which Fe^3+^ and Fe^2+^ cations are simultaneously present, with half of the Fe^3+^ population occupying the tetrahedral sites, and the other half the octahedral sites together with the remaining Fe^2+^ cations. An ‘intermediate’ of these two iron oxides that has also been studied comparatively less is maghemite (γ-Fe_2_O_3_) [32,33]. The latter can be indeed considered a polymorph of α-Fe_2_O_3_ in terms of chemical formula, yet it exhibits structural properties closely related to Fe_3_O_4_, constituting effectively a Fe(II)-deficient magnetite with associated vacancies.

Room temperature chemical and electrochemical insertion of alkali ions into α-Fe_2_O_3_ and Fe_3_O_4_ has been pioneered since the 1980s. A first structural study [34] established a compositional range of 0 < x < 2 for lithium insertion in these oxides (i.e., Li_x_Fe_2_O_3_ and Li_x_Fe_3_O_4_) highlighting their respective structural changes during Li incorporation and reporting that additional lithiation (x ≥ 2) resulted in irreversible phase separation through the formation of metallic Fe and Li_2_O (i.e., conversion). This mechanism was later investigated in detail not only for iron oxides, but also for a series of other transition metal oxides (TMOs) [35], showing that high capacities were accessible through this type of conversion reaction, which differs from both ion insertion and Li-alloying. The conversion reactions of most TMOs appeared surprisingly reversible compared to those of other metal oxides (e.g., SnO, SnO_2_, Sb_2_O_3_, etc., that undergo further Li-alloying), although they exhibit a remarkable polarization between their discharge/charge curves. A general scheme of reaction was proposed for conversion/de-conversion of TMOs via uptake and removal of Li [12] according to:

[TM]_a_O_b_ + (2b)·Li^+^ + (2b)·e^−^ → a·[TM]^0^ + b·Li_2_O (1)

a·[TM]^0^ + b·Li_2_O ↔ (2b)·Li^+^ + (2b)·e^−^ + [TM]_a_O_b_^*^ (2)

where TM represents the transition metal (e.g., Fe, Co, Ni, Cu, Mn, etc.) and initial lithiation in Equation (1) causes an irreversible structural transformation of the pristine TMO into a nanocomposite made of small metal nanoparticles embedded in an amorphous Li_2_O matrix. Lithium can subsequently be removed from the TM/Li_2_O nanocomposite via the reversible reaction in Equation (2), where the asterisk on the right-hand side indicates that the de-converted oxide cannot return to its pristine state, while it retains nano-sized features dictated by the original formation of the metal nanoparticles and extensive phase boundaries with the surrounding Li_2_O. Contrary to ion insertion, which often results in reversible structural changes to the host lattice—especially for limited ion amounts—and can thus support topotactic mechanisms (e.g., Li intercalation in graphite), conversion belongs to a class of reconstitution reactions with a distinctive atomic displacement character, in which the elements forming the compounds are irreversibly set apart, creating phase boundaries.

One associated relevant aspect is that in standard, non-aqueous carbonate-based electrolytes the conversion reaction in Equation (1) is accompanied by SEI formation roughly below 1.0 V vs. Li^+^/Li, which typically accounts for limited Coulombic efficiency in the first cycle. This represents a critical factor, given extensive interfacial areas naturally involved in the oxide conversion, and the formation of small (<5 nm) metallic nanoparticles with a high surface-to-volume ratio.

Another feature associated with the conversion mechanism is a distinctive pseudo-capacitive charge storage occurring at low voltages vs. Li^+^/Li, which contributes to a substantial ‘extra capacity’, thus exceeding the theoretical charge value. The origin of this extra capacity has been a matter of intense debate according to different models [36,37,38] and recently has been demonstrated via in situ magnetometry to be related to a space charge mechanism, through which spin-polarized e^−^ are localized on the metal nanoparticles’ surface, creating a strong surface capacitance [39] together with Li^+^ simultaneously located at the neighboring Li_2_O interface. This additional charge storage contribution at the nanoscale is intriguing, as it can boost power delivery in a way similar to that of supercapacitors and could effectively constitute a bridge between batteries and capacitors [40]. Moreover, given the nano-sized characteristics of such TM/Li_2_O composites and the possibility of tailoring beforehand the size and shape of the pristine TMO (nano)particles/structures, it should be possible to further influence this pseudo-capacitive behavior. In this way, one could control more effectively not only the extent of the conversion reaction and favor its kinetics, for example, through reduced dimensions of the TMO, but also the total interface involved in such capacitive charge storage mechanisms.

Using a nanostructured approach, through which the TMO is formed in desired nanostructures with adjustable size, shape, composition and uniform size distribution, enables more homogeneous reactions and renders the active material more mechanically robust upon repeated volume variations during cycling (e.g., conversion/de-conversion). Increased mechanical stability is associated with convenient accommodation of the volume changes and reduced tendency toward fracture, stemming from an enhanced surface-to-volume ratio for the constitutive atoms in the nanomaterial. This aspect is particularly relevant for the conversion/de-conversion of iron oxides, as they typically undergo noticeable volume changes upon Li uptake/removal (e.g., ≈96% for α-Fe_2_O_3_ [41], ≈82% for Fe_3_O_4_ [42]).

Ensuring continuous electron percolation and effective ion transport through coated electrodes containing iron oxides as possible anodes is paramount for a reliable functioning of the corresponding cells. Designing the structure of the active material and/or the electrode functionalities can play additional roles in extending the performances and cycleability of these iron oxides, partly compensating for their intrinsic shortcomings.

### 5.2. Design of Composite Iron Oxide/Carbon Nanostructures for LIBs

Realization of hybrid structures composed of carbonaceous materials and nanostructured iron oxide, for example, provides a valid route to establish and preserve the above functionalities. These structures have emerged as one promising option for reaching an ideal trade-off among thermal/chemical stability, capacity and rate capability. On the one hand, the presence of the carbon material as a coating or enveloping layer contributes to enhanced electronic conductivity, mitigates the impact of volume expansion/contraction during cycling, reduces the aggregation of the iron oxides upon repeated discharge/charge and prevents direct contact of the reacting material with the electrolyte, thus inducing a better electrochemical performance. On the other hand, the nanostructures shorten the Li^+^ and electron transport distances and enlarge the electrode–electrolyte contact areas, providing faster reactions and accommodating effectively the structural strain generated during the Li^+^ incorporation/release processes [43].

**Figure 3:**
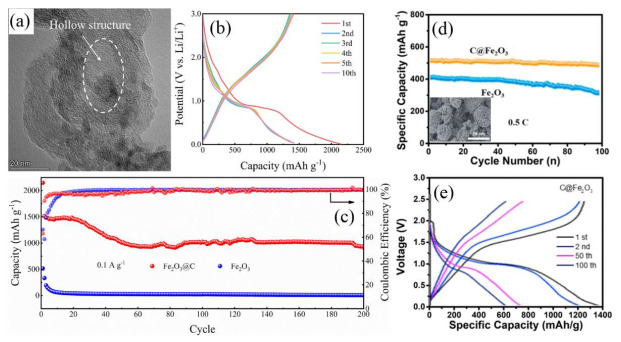
(**a**) Transmission electron microscopy (TEM) image of Fe_2_O_3_@C composite, highlighting its local hollow structure [14]. The scale bar is 20 nm. (**b**) Galvanostatic voltage profiles of discharge–charge for some selected cycles of Fe_2_O_3_@C composite obtained between 0.01 V and 3.00 V vs. Li^+^/Li at 0.1 A g^−1^ [14]. (**c**) Comparison of capacities and Coulombic efficiencies of Fe_2_O_3_ and Fe_2_O_3_@C, respectively, cycled at 0.1 A g^−1^ over 200 galvanostatic cycles [14]. (**d**) Capacity retention over 100 cycles for hierarchical C@Fe_2_O_3_ hybrid structure and pure Fe_2_O_3_ [45] cycled at 0.5 C, respectively. The inset shows a scanning electron microscopy (SEM) of the hierarchical C@Fe_2_O_3_ hybrid structure and its scale bar is 50 nm. (**e**) Galvanostatic discharge–charge curves for a number of selected cycles for the hierarchical C@Fe_2_O_3_ hybrid structure cycled between 0.01 and 2.50 vs. Li^+^/Li at 0.1 C [45]. Reprinted with permission from Ref. [14]. Copyright 2022 Elsevier. and Ref. [45]. Copyright 2022 Springer Nature.

In this direction, yolk-shell Fe_2_O_3_@C nanospheres (Figure 3a) had as a result a good cyclic stability retaining a capacity value of 929 mAh g^−1^ after 200 cycles at 0.1 A g^−1^ (Figure 3b,c) [14]. The role of carbon black encapsulating the Fe_2_O_3_ nanoparticles and creating local voids around them was crucial to buffering effectively their volume changes during the Li^+^ uptake/removal processes and enhancing the electronic/ionic conductivity of the oxide. Below, one can see the possible reactions taking place with initial insertion of Li into Fe_2_O_3_ in Equation (3), subsequent irreversible reduction of Li_2_(Fe_2_O_3_) into metallic Fe and amorphous Li_2_O yielding conversion in Equation (4) and oxidation of metallic Fe to Fe_2_O_3_ during the de-conversion step in Equation (5) [44].

Fe_2_O_3_ + 2Li^+^ + 2e^−^ → Li_2_(Fe_2_O_3_) (3)

Li_2_(Fe_2_O_3_) + 4Li^+^ + 4e^−^ → 2Fe^0^ + 3Li_2_O (4)

2Fe^0^ + 3Li_2_O ↔ Fe_2_O_3_ + 6Li^+^ + 6e^−^ (5)

Zhao et al. reported an in situ loading of Fe_2_O_3_ nanoparticles on carbon spheres arising in a hybrid hierarchical structure with a capacity of 1386 mAh g^−1^ maintaining a value of 602 mAh g^−1^ after 100 cycles [45], see Figure 3d,e.

**Figure 4:**
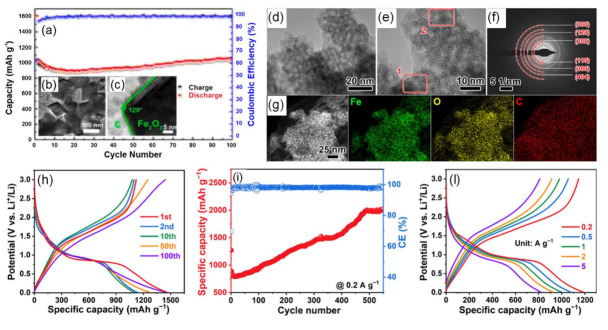
(**a**) Evolution of discharge–charge capacities and Coulombic efficiency for 100 galvanostatic cycles of Fe_2_O_3_@C composite with octahedron-like Fe_2_O_3_ particles [46]. The Fe_2_O_3_@C composite was cycled at 0.2 A g^−1^ between 0.02 V and 3.00 V vs. Li^+^/Li. The octahedron-like shape of Fe_2_O_3_ is highlighted in the inset showing two transmission electron microscopy (TEM) images (**b**,**c**) at low and high resolution of the Fe_2_O_3_@C composite, with respective scale bars of 100 nm (**b**) and 5 nm (**c**). (**d**,**e**) TEM images of iron oxide/carbon composite with ultrafine Fe_2_O_3_ nanocrystals anchored on reduced graphene oxide (rGO) [47] and (**f**) associated selected area electron diffraction (SAED). (**g**) TEM image and related X-ray energy dispersive (EDX) analyses of the Fe_2_O_3_@C composite showing Fe (green), O (yellow) and C (red) elemental maps, respectively. The scale bar in (**g**) is 25 nm. (**h**) Galvanostatic discharge–charge profiles for some selected cycles of Fe_2_O_3_@C at 0.2 A g^−1^ in a potential range of 0.01–3.00 V vs. Li^+^/Li. Note a capacity increase and a variation of the slope of the discharge–charge curves after 100 cycles. (**i**) Cycling performances of the Fe_2_O_3_@C nanocomposite at 0.2 A g^−1^ showing its capacity evolution and Coulombic efficiency over 550 cycles [48]. (**l**) Galvanostatic profiles of charge–discharge of Fe_2_O_3_@C obtained in correspondence with different current densities. Note that a high capacity of ≈810 mAh g^−1^ was still accessible during cycling at 5.0 A g^−1^. Reprinted with permission from Refs. [46,47]. Copyright 2022 Elsevier.

In another work [46], octahedron-like Fe_2_O_3_ was embedded in carbon (Fe_2_O_3_@C), facilitating fast charge transport. This was due to a uniform distribution of monodisperse octahedron-like Fe_2_O_3_ nanoparticles on the carbon framework (Figure 4b,c), which reduced the ion diffusion distance. Consequently, the composite delivered a high rate capability with a capacity of 414 mAh g^−1^ at 5.0 A g^−1^, a moderately rising capacity around the 20th cycle yielding 1025 mAh g^−1^ after 100 cycles at 0.2 A g^−1^ (Figure 4a) and a superb cycling ability with a capacity of retention of 82.1% after 600 cycles at 1.0 A g^−1^. A very recent publication indicated that Fe_2_O_3_ nanocrystals provide abundant electroactive edge sites on reduced graphene oxide (rGO) nanosheets (Figure 4d–g), buffering the volume changes [47]. This had as an outcome a large discharge capacity of 1462 mAh g^−1^ and a following charge capacity of 1121 mAh g^−1^ with an initial Coulombic efficiency of 76.7% (Figure 4h). These Fe_2_O_3_ nanocrystals/rGO electrodes exhibited an excellent cycleability at 0.2 A g^−1^ with rising capacities reaching values above 2000 mAh g^−1^ after 550 cycles (Figure 4i), as well as elevated capacity retention upon application of increasing gravimetric current densities (Figure 4l), which enabled a capacity value of 993 mAh g^−1^ after 500 cycles at 1.0 A g^−1^. This phenomenon of anomalous rising capacity (also termed ‘negative fading’) upon progressive cycling is certainly intriguing and, although earlier observed in different TMOs cycled in various types of electrode structures under distinctive conditions [47,48], its origin is still rather obscure and controversial [49]. Three main different explanations have been proposed so far for this anomalous capacity rise [50], namely: (i) Enhanced utilization of the conversion/de-conversion reactions thanks to a sensibly reduced size of the active particles, which leads to the creation of new electrochemically active sites generating such capacity increase; (ii) optimization of their electrolyte-derived interface layers linked to a catalytic effect of the metallic nanoparticles formed during the conversion, through which an extensive contact area between the active nanomaterials and the electrolyte is established, promoting the growth of such surface layers and thus the capacity; (iii) changes in the morphology of the active materials related to sharp variations of their porosity and/or enhancement of their surface area, which both account for an improved reaction kinetics through a facilitated transport of the charge carriers boosting the resulting capacity.

The abovementioned results clearly confirm a series of significant advantages of employing hybrid nanostructures (see later Table 1) to promote the ion and electron transport, concurrently inhibiting associated volume changes occurring during the cycles, thanks to their high surface-to-volume ratio that renders these structures more mechanically resilient and less prone to undergo fracturing or crumbling. Regarding nano-engineering of Fe_2_O_3_, nanocrystals—as compared with other structures—could accommodate effectively the mechanical strain during the cycling process, maximizing the beneficial roles of both Fe_2_O_3_ and the carbon material.

Another important matter of consideration for future perspectives is related with the manufacturing process of such active nanomaterials with tailored functionalities [43], which is a key factor to boost the rapid transition of batteries towards mass production. Up to the present, procedures including various steps are presented for the development of these hybrid structures facing a number of limitations such as possible contamination, lack of reproducibility in large areas and/or low growth rates. Therefore, a deep understanding of the manufacturing processes is an equally important challenge to guarantee a high throughput and flexibility in their production phase together with the highest quality, safety and performance at the cell level.

Magnetite (Fe_3_O_4_) is another iron oxide for possible use in LIBs due to its interesting characteristics (i.e., high theoretical capacity, environmental benignity, low cost, moderate electrical conductivity, magnetic characteristics [51]). Nanostructuring along with the inclusion of carbon materials have also been applied to address the irreversible capacity losses and linked degradation of the capacity retention of Fe_3_O_4_ upon extensive cycling (see Table 1).

**Figure 5:**
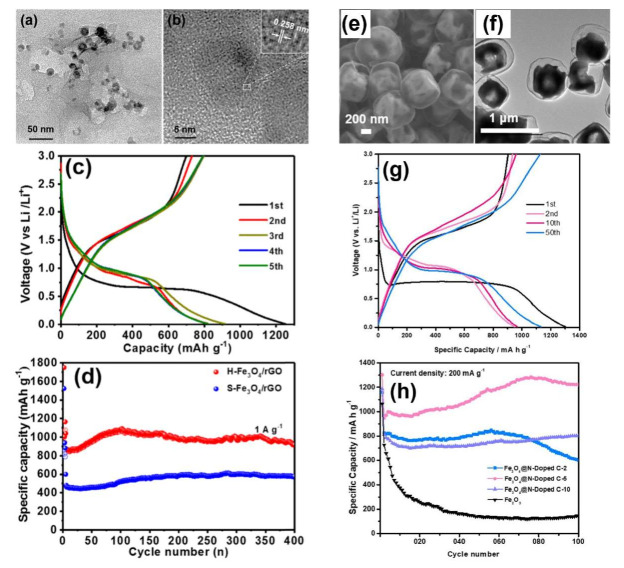
(**a**) Low-resolution transmission electron microscopy (TEM) image of hollow Fe_3_O_4_ anchored on reduced graphene oxide (rGO) [15] with a scale bar of 50 nm. (**b**) High-resolution TEM (HRTEM) micrograph showing a detail of the H-Fe_3_O_4_/rGO nanocomposite and its lattice fringes in the inset; the scale bar in (**b**) is 5 nm. (**c**) Galvanostatic voltage profiles of discharge/charge for some selected cycles of H-Fe_3_O_4_/rGO obtained at 0.5 A g^−1^ between 0.01 and 3.00 V vs. Li^+^/Li. (**d**) Long-term cycle performance of H-Fe_3_O_4_/rGO and comparison with that of solid-Fe_3_O_4_/rGO upon application of 1.0 A g^−1^ [15]. (**e**) SEM image of hollow core-shell Fe_3_O_4_/N-doped C nanocomposite (Fe_3_O_4_@void@N-doped C-5), the scale bar is 200 nm [52], adapted from [52]. (**f**) TEM image of Fe_3_O_4_@void@N-doped C-5 with a scale bar of 1 μm. (**g**) Galvanostatic voltage profiles of discharge/charge at 0.2 A g^−1^ for Fe_3_O_4_@void@N-doped C-5 showing some selected cycles obtained in the potential range of 0.01–3.00 V vs. Li^+^/Li. (**h**) Comparison of cycle performances for a series of hollow core-shell Fe_3_O_4_/N-doped C nanocomposites obtained at 0.2 A g^−1^ showing that capacities higher than 1000 mAh g^−1^ could be obtained via Fe_3_O_4_@N-doped C-5 after 100 cycles [52]. Reprinted with permission from Ref. [15]. Copyright 2022 Elsevier.

A silkworm-chrysalisnet-like Fe_3_O_4_/rGO presented a capacity of 916.4 mAh g^−1^ after 100 cycles at 0.5 A g^−1^ [51]. This research work managed to disperse the Fe_3_O_4_ nanostructures uniformly on rGO due to electronegative oxygen functional groups of rGO, preventing particle agglomeration during their continuous charge/discharge cycles. By utilizing the same carbon medium, it was found that the hollow Fe_3_O_4_/rGO composite (Figure 5a,b) presented an initial discharge capacity value of 1251 mAh g^−1^ (Figure 5c) with a capacity retention of 56.7% compared to its solid Fe_3_O_4_/rGO counterpart [15]. The performances of hollow Fe_3_O_4_/rGO (H-Fe_3_O_4_/rGO) and solid Fe_3_O_4_/rGO (S-Fe_3_O_4_/rGO) were compared, revealing the superiority of the hollow structures in capacity, rate capability and stability (Figure 5d). Wang et al. indicated that hollow core-shell Fe_3_O_4_/N-doped C nanocomposites (Figure 5e,f) could reach a value of 1222 mAh g^−1^ at 200 mA g^−1^ after 100 cycles (Figure 5g,h) [52]. In this case, the critical parameter was the void size between the carbon shell and the Fe_3_O_4_ to accommodate the volume enlargement upon lithium uptake and enhance the Li^+^ diffusion.

**Figure 6:**
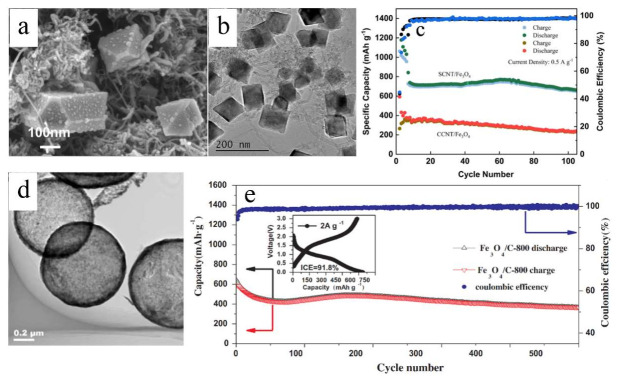
(**a**) Scanning electron microscopy (SEM) image and (**b**) low magnification transmission electron microscopy (TEM) of SCNT/Fe_3_O_4_ [35]. (**c**) Specific capacity and Coulombic efficiency of SCNT/Fe_3_O_4_ and CCNT/Fe_3_O_4_ at 0.5 A g^−1^ as a function of cycle number [54]. (**d**) TEM image of hollow Fe_3_O_4_/C microspheres via ultrasonic spray pyrolysis at 800 °C [56]. (**e**) Specific capacity and Coulombic efficiency of hollow Fe_3_O_4_/C-800 microspheres upon long-term galvanostatic cycling at a current density of 2.0 A g^−1^. The inset shows the voltage profiles of discharge and charge for the first cycle [56]. Reprinted with permission from Ref. [35]. Copyright 2022 Nature and Refs. [54,56]. Copyright 2022 Elsevier.

Carbon nanotubes (CNTs) were also utilized in hybrid composites due to their structural stability [53,54]. Nevertheless, they present chemical inertness and low hydrophilicity, which makes it difficult to disperse the Fe_3_O_4_. In order to deal with this drawback, acidization (CCNT) and sulfonation (SCNT) of CNTs were separately carried out, indicating that the Fe_3_O_4_ nanoparticles immersed in sulfonated carbon nanotubes (SCNTs) possessed a reversible capacity of 674 mAh g^−1^ at 500 mA g^−1^ after 100 cycles (Figure 6c) [54]. Only the SCNTs were able to distribute the sulfonic acid groups on their surfaces, which were used as nucleation sites of Fe reactant for the preparation of SCNT/Fe_3_O_4_ (i.e., polyhedral structure twisted with tubular features) (Figure 6a,b). On the other hand, the acidization caused serious damage to the resulting structure and its electrical conductivity, causing inability to form a stable composite with Fe_3_O_4_ [54].

Hence, it is clear that also for Fe_3_O_4_ a resilient hybrid structure with carbon is necessary to support its electrochemical reactions with Li during respective insertion, conversion and de-conversion according to a detailed understanding as one can see in Equations (6)–(9) [55]:

Fe_3_O_4_ + xLi^+^ + xe^−^ → Li_x_Fe_3_O_4_ with 0 < x ≤ 2 (6)

Li_2_Fe_3_O_4_ + 2Li^+^ + 2e^−^ → 2FeO·Li_2_O + Fe^0^ (7)

2FeO·Li_2_O + Fe^0^ + 4Li^+^ + 4e^−^ → 3Fe^0^ + 4Li_2_O (8)

3Fe^0^ + 4Li_2_O ↔ Fe_3_O_4_ + 8Li^+^ + 8e^−^ (9)

although Equation (9) could possibly be replaced by another step:

3Fe^0^ + 4Li_2_O ↔ 3FeO + Li_2_O + 6Li^+^ + 6e^−^ (10)

where the de-lithiated phase should be considered to have a FeO-type structure, yet with lithium presumably incorporated in it [55]. As a result of such stark structural changes and amorphous-like nature of the Fe^0^/Li_2_O nanocomposite, the initial moderate electronic conductivity of Fe_3_O_4_ (σ_el_ ≈ 2 × 10^2^ S cm^−1^) is not retained and thus applying a conductive carbon shell, providing an effective local electrical wiring to the reacting particles, is required to ensure long-lasting cycling of this active material.

Once again, manufacturability, feature control and possibility for upscaling of controlled syntheses of hybrid Fe_3_O_4_/C nanostructures with minimal agglomeration, possibly including voids to buffer the volume variations, are key factors in view of a possible large-scale production. A study reported, for example, a one-step rapid preparation of hollow Fe_3_O_4_/C microspheres through a continuous process employing an ultrasonic spray pyrolysis route [56] that enabled direct scalability and good control over the resulting materials and their porosity (Figure 6d). The latter offered good cycle performances for the Fe_3_O_4_/C microspheres synthesized at 800 °C, with a reversible capacity of about 600 mAh g^−1^ after 200 cycles at 1.0 A g^−1^ and long-term cycling at 2.0 A g^−1^ providing capacities close to 400 mAh g^−1^ after 500 cycles (Figure 6e).

**Table 1:** Synoptic comparison of the electrochemical performances obtained in Li-ion cells for some types of hybrid nanostructures containing iron oxides and carbonaceous materials. These active nanomaterials were investigated as potential anodes for LIBs and typically cycled between 0.01 and 3.0 V vs. Li^+^/Li.

Anode	Electrolyte	Capacity	Stability
yolk-shell Fe_2_O_3_@C nanospheres [14]	1 M LiPF_6_ 1:1:1 (*v*/*v*/*v*) of ethylene carbonate (EC)/diethyl carbonate (DEC)/ethyl methyl carbonate (EMC)	929 mAh g^−1^ at 0.1 A g^−1^	200 cycles
Fe_2_O_3_@C [46]	-//-	756 mAh g^−1^ at 1 A g^−1^	82.1 % retention after 600 cycles
Hierarchical structure of C@Fe_2_O_3_ [45]	-//-	1386 mAh g^−1^ at 0.1 C	602 mAh g^−1^ after 100 cycles
Nanocrystals Fe_2_O_3_@rGO [47]	-//-	1175 mAh g^−1^ at 0.2 A g^−1^	993 mAh g^−1^ after 500 cycles at 1 A g^−1^
Silkworm-chrysalisnet-like Fe_3_O_4_/rGO [51]	-//-	916.4 mAh g^−1^ at 0.5 A g^−1^	100 cycles
Hollow Fe_3_O_4_/rGO [15]	-//-	827.3 mAh g^−1^ at 0.5 A g^−1^	550 cycles
Hollow Fe_3_O_4_/rGO [52]	-	1222 mAh g^−1^ at 200 mA g^−1^	100 cycles
SCNTs/Fe_3_O_4_ [54]	-//-	674 mAh g^−1^ at 500 mA g^−1^	100 cycles
Hollow Fe_3_O_4_/C microspheres [56]	-//-	≈ 600 mAh g^−1^ at 1.0 A g^−1^	200 cycles

A rational design of these hybrid iron oxide/carbon nanostructures with built-in voids certainly helps in improving both local electrical wiring and Li-ion conduction, while addressing suitably the challenges of repeated volume variations and particle agglomeration with linked SEI growth. This allows prolonging significantly the cycle life of the corresponding cells through an improvement of the Coulombic efficiency and overall performances. However, two additional key limitations of iron oxides, when it comes to their effective use as LIB anodes, cannot still be overcome by this approach, namely: (i) Their scarce energy efficiency (i.e., round-trip efficiency) due to a remarkable voltage hysteresis in each charge/discharge cycle and (ii) their inconvenient operating voltage window for capacity delivery. While many attempts have been made, these drawbacks cannot be solved through a sheer materials engineering approach, apparently. They rather represent intrinsic aspects unavoidably associated with the conversion/de-conversion mechanism and repeated formation/dissolution of metallic nanoparticles involving large interface energy changes [57,58], although the voltage hysteresis of iron fluoride has also been ascribed to possible kinetics limitations [59]. This crucial point of voltage hysteresis and energy losses can be mitigated only partly by aimed active materials/interface modifications [60,61], or circumvented by deliberately operating the converted iron oxides mainly in their reversible pseudo-capacitive charge storage regime in full cells, thus avoiding cyclic conversion/de-conversion [62,63], simultaneously restricting the voltage range to rather low potentials vs. Li^+^/Li, yet sacrificing most of the capacity.

This dilemma with either accepting poor energy efficiencies or limited capacities (and energy densities) represents likely a central knot in possible future utilization of iron oxides in LIBs, as these hybrid materials have attractive features, yet their use and ultimate energy storage performances are not on par with those of graphite, certainly in terms of Coulombic and energy efficiencies. Besides, the actual energy density that can be obtained with such hybrid iron oxide/carbon nanostructures can be limiting, especially when their tap density is much lower than that of graphite and their built-in empty space to accommodate the volume changes is taken into account as well. Nonetheless, long-term cycling of these hybrid iron oxide/carbon structures is clearly possible and their performances have progressively been improving after their early discovery. These facts alone can be certainly relevant for potential future applications in which the energy efficiency would not be a stringent criterion, while their mixed faradic and capacitive charge storage could be deemed not only as a highly desirable characteristic, but also as a real prerequisite.

The conversion of iron oxides into Fe^0^/Li_2_O by extensive lithiation is fundamentally intriguing, as the storage of Li^+^ and e^−^ occurs in a rather ‘compact’ way through this mechanism. In fact, it allows to form in situ a highly divided nanocomposite in which the two component phases (and their interfaces) are packed rather densely, while Li_2_O possesses intrinsically very high theoretical gravimetric and volumetric energy densities due to its high lithium content in the presence of only very light elements. The facts that (i) Fe^0^ nanoparticles and surrounding Li_2_O can concurrently be formed, (ii) the lithium-storing phase is effectively the oxygen present in the original oxide, while (iii) Fe does not alloy with Li, make this nanocomposite highly dependent on the physicochemical characteristics of these two nano-phases (i.e., Fe^0^, Li_2_O) as well as possible intermediates (i.e., FeO·Li_2_O) for its electrochemical behavior. This observation is relevant here, as changing the type of ion used in the electrochemical processes to yield progressive insertion and possible conversion of the iron oxides has unavoidably profound effects. It can be anticipated that electrochemical lithiation, sodiation and potassiation of iron oxides typically retain similarities only in some general aspects, while their actual characteristics and performances are particularly distinctive.

### 5.3. Design of Composite Nanostructures through Iron Oxide-Based Materials and Carbon for KIBs

Electrochemical reaction of iron oxides with K^+^ for potential development of future KIBs has hardly been reported so far, contrary to Na^+^ ions, for which an increasing number of NIB investigations exists already [64,65,66].

An initial study on tentative electrochemical reactions of K^+^ with a nanostructured composite material made of iron(III) oxyhydroxide and iron(VI) oxide showed the possibility of reversible insertion/de-insertion of different alkali ions (K^+^, Na^+^ and Li^+^) into such a compound [67]. The range of potentials investigated for the corresponding half-cells was mainly directed toward a cathode application and highlighted significant capacities, although with rather limited cycling stabilities and a clear presence of side reactions, especially during the first cycles. This undesirable feature was particularly marked for the K half-cell, during the first discharge to 1.0 V vs. K^+^/K, and likely due to electrolyte decomposition with possible SEI formation. The discharge capacities of the various half-cells obtained at a current density of 0.05 mA cm^−2^ after the first cycle were 320 mA h g^−1^ with Li^+^, 218 mA h g^−1^ with Na^+^ and 150 mA h g^−1^ with K^+^, respectively. A minor tendency toward ion insertion and associated available capacity was noticed for the Na and K half-cells compared to their Li counterpart. This discrepancy was explained mainly in terms of increasing ionic radius for the corresponding guest ions, which had an impact on their diffusion properties and thus related kinetics of the respective reactions in these different types of half-cells.

It is interesting to note that even without entering a regime for iron oxide conversion, which typically begins below ≈0.9 V vs. Li^+^/Li, the difference in size of these alkali ions—especially that existing between Li^+^ (0.76 Å) and K^+^ (1.38 Å)—plays a clear role in the electrochemical behavior of the corresponding half-cells. Hence, if the insertion mechanism of iron oxide appears already quite influenced by the size of the guest ion (see also Table 2), it can reasonably be expected that its following conversion can be even more deeply affected by this factor. In fact, an ideal conversion of iron oxide through incorporation of potassium in principle should yield:

Fe_x_O_y_ + (2y)·K^+^ + (2y)·e^−^ → x·Fe^0^ + y·K_2_O (11)

x·Fe^0^ + y·K_2_O ↔ (2y)·K^+^ + (2y)·e^−^ + Fe_x_O_y_^*^ (12)

similarly to the abovementioned reactions of TMOs with lithium in Equations (1) and (2). However, this is only in theory, while a possible formation of the Fe^0^ and K_2_O nano-phases most likely displays in reality a different behavior compared to its Fe^0^ and Li_2_O counterparts. This can be rationalized in terms of different ionic sizes, electrochemical redox potentials, as well as distinctive molar volumes for the generated alkali oxides that, in turn, could possess dissimilar ion conductivities. As an immediate consequence, the theoretical volume variations involved in such reactions are much more severe than those brought about by lithiation (see Table 3).

**Figure 7:**
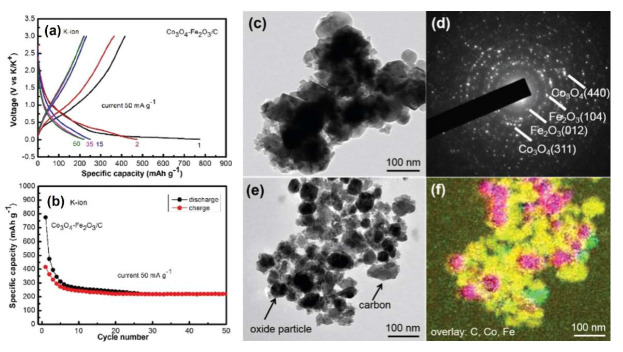
(**a**) Galvanostatic voltage profiles of charge/discharge for a number of selected cycles for a hybrid Co_3_O_4_–Fe_2_O_3_/C material cycled in a K half-cell at 50 mA g^−1^ between 0.01 and 3.00 V vs. K^+^/K. (**b**) Cycle performance and capacity retention of the Co_3_O_4_–Fe_2_O_3_/C for the same K half-cell. (**c**) Bright-field transmission electron microscopy (TEM) of the Co_3_O_4_–Fe_2_O_3_ material and (**d**) the corresponding selected area electron diffraction (SAED) pattern for this compound showing its nano-crystalline nature. (**e**) Bright-field TEM image of the hybrid Co_3_O_4_–Fe_2_O_3_/C together with (**f**) an overlay of chemical maps for the region displayed in (**e**) showing elemental Co, Fe and C maps. (Colour scheme: Cobalt, green; iron, red; carbon, yellow). Note the prominent capacity loss in the first cycle in (**a**) and (**b**), as well as the discrepancies between the discharge and charge capacities in (**b**) during the first 25 cycles. Reprinted with permission from Ref. [16]. Copyright 2022 Royal Society of Chemistry.

An early study attempting the use of conversion reactions of iron oxide [16,75] to develop KIB anodes took this key point of exacerbated volume changes upon potassiation into account and devised a hybridization of inter-dispersed Co_3_O_4_ and Fe_2_O_3_ nanoparticles in a carbon matrix (Figure 7-right panel) via a gentle ball-milling approach to address this issue.

This strategy provided a buffering action against the volume variations via mixed conversion reactions of the two TMOs with K^+^ and the surrounding carbon improving the electrochemical behavior of the electrodes. The hybrid Co_3_O_4_–Fe_2_O_3_/C nanomaterial displayed a moderate electrochemical activity toward K^+^, yielding a reversible capacity of about 220 mAh g^−1^ at 50 mA g^−1^ (Figure 7a,b) after 50 cycles, although a pronounced irreversible charge loss occurred in the first cycle, likely due to electrolyte decomposition and SEI formation. In the second cycle, only a limited capacity was accessible and its values were slightly above 400 mAh g^−1^, fading rapidly during the first 10 cycles to ≈250 mAh g^−1^. Ex situ x-ray diffraction (XRD) analysis of the cycled electrode after full potassiation exhibited characteristic diffraction peaks for Co, Fe and K_2_O, thus supporting the hypothesis that Co_3_O_4_ and Fe_2_O_3_ reacted through a conversion mechanism similar to that reported in Equations (1) and (2), in which the role of Li^+^ is taken on by K^+^. Nevertheless, the characteristic peaks of both Co_3_O_4_ and Fe_2_O_3_ were also visible in the diffraction pattern of the potassiated electrode to 0.01 V vs. K^+^/K, thus demonstrating that the Co_3_O_4_–Fe_2_O_3_/C did not undergo completive conversion upon extensive potassiation. The Co_3_O_4_ and Fe_2_O_3_ diffractions were also observed after de-potassiation of the electrode at 3.0 V vs. K^+^/K, although the Co and K_2_O conversion products were still detected in the diffractogram, suggesting that the conversion mechanism was not complete and only partially reversible [16]. A higher extent of conversion appeared accessible through subsequent cycling of the electrode, since after the 10th discharge at 0.01 V vs. K^+^/K all the Co_3_O_4_ and Fe_2_O_3_ diffractions disappeared almost completely. Only a limited capacity was available through this nanostructured-hybrid approach, which nonetheless highlighted the crucial role of the carbon matrix in supporting the electrochemical reactions.

**Figure 8:**
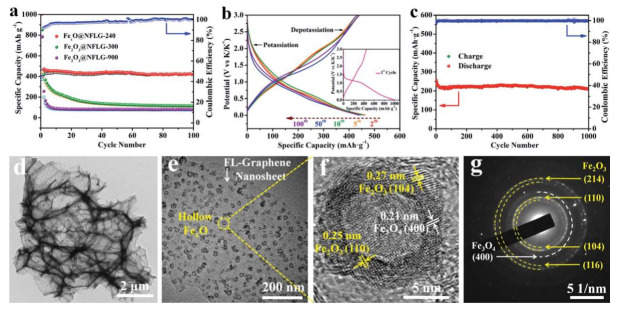
(**a**) Comparison of cycle performances and capacity retention for Fe_x_O@NFLG-240, Fe_2_O_3_@NFLG-300 and Fe_2_O_3_@NFLG-900 during 100 discharge/charge cycles carried out at a constant gravimetric current density of 50 mA g^−1^ between 0.01 and 3.00 V vs. K^+^/K. The Coulombic efficiency data points in (**a**) refer to the cycles of Fe_x_O@NFLG-240. (**b**) Galvanostatic discharge/charge voltage profiles for a number of selected cycles for Fe_x_O@NFLG-240 obtained under the above conditions. The inset in (**b**) presents the voltage profiles of Fe_x_O@NFLG-240 during the first cycle, highlighting a severe capacity loss likely due to SEI formation. (**c**) Long-term cycle performances and capacity retention of Fe_x_O@NFLG-240 under galvanostatic cycling at 2.0 A g^−1^. (**d**) Low-magnification transmission electron microscopy (TEM) of Fe_x_O@NFLG-240. (**e**) TEM image of homogeneously distributed hollow Fe_x_O nanospheres anchored on N-doped few layer graphene (NFLG). (**f**) HR-TEM close-up image of a hollow Fe_x_O nanosphere indicated in (**e**) displaying characteristic crystal lattice fringes for respective crystallographic planes of Fe_2_O_3_ and Fe_3_O_4_ and (**g**) associated selected area electron diffraction (SAED) pattern highlighting the simultaneous presence of these distinctive nano-crystalline oxide phases. Reprinted with permission from Ref. [75]. Copyright 2022 Royal Society of Chemistry.

Another example of hybrid nanostructured iron oxide/carbon material for use as possible KIB anode through conversion-type reactions is represented by a composite constituted of hollow Fe_x_O nanospheres anchored on 3D N-doped few-layer graphene, abbreviated as Fe_x_O@NFLG [75]. This type of functional active material was synthesized via a potentially scalable chemical bubbling route combined with further annealing at 900 °C under Ar and then cooled to 240 °C to produce a corresponding Fe_x_O@NFLG-240 nanocomposite (Figure 8, lower row).

This approach aimed at providing significant surrounding space to alleviate the volume variations of the hollow Fe_x_O spheres during extensive potassiation/de-potassiation, while achieving a uniform distribution and anchoring of the latter on NFLG. The Fe_x_O@NFLG-240 provided a high capacity of ≈1000 mAh g^−1^ during the first discharge in a K half-cell cycled at 50 mA g^−1^, yet only a limited charge capacity of ≈450 mAh g^−1^ in the next half-cycle, likely due to irreversible electrolyte consumption and SEI layer formation (see inset of Figure 8b). After the first cycle, the galvanostatic discharge–charge profiles appeared rather consistent and stable capacities of ≈420 mAh g^−1^ were obtained over 100 cycles at the same current density (Figure 8a), validating the effectiveness of this strategy. This allowed prolonged cycling of the K half-cells with Fe_x_O@NFLG-240 that could cycle for 1000 cycles at 2.0 A g^−1^ retaining a moderate capacity of about 200 mAh g^−1^ (Figure 8c) and even withstand 5000 cycles at 5.0 A g^−1^ with capacities around 140 mAh g^−1^.

**Figure 9:**
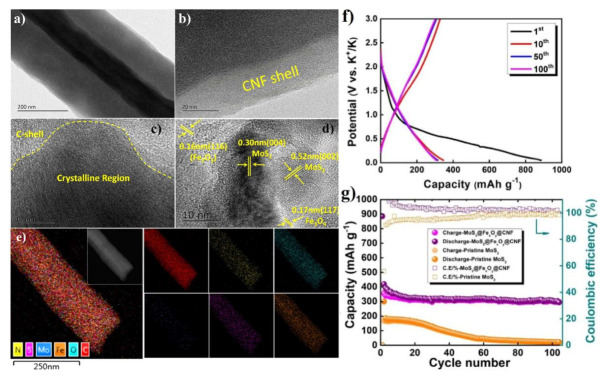
(**a**) Low magnification transmission electron microscopy (TEM) of an electrospun core-shell MoS_2_@Fe_x_O_y_@CNF. (**b**) High resolution TEM (HRTEM) image of the MoS_2_@Fe_x_O_y_@CNF and (**c**) HRTEM image highlighting the contour between a crystalline region of the core and amorphous surrounding CNF shell. (**d**) HRTEM close-up image showing a series of lattice fringes assigned to different characteristic crystalline planes of Fe_2_O_3_, Fe_3_O_4_ and MoS_2_, respectively. (**e**) Overlay of EDX elemental maps for MoS_2_@Fe_x_O_y_@CNF in correspondence with its topographic image displayed in the inset. Associated individual elemental maps for C (red), N (yellow), O (cyan), Mo (blue), S (violet) and Fe (orange), respectively. (**f**) Galvanostatic discharge/charge voltage profiles in correspondence with some selected cycles for MoS_2_@Fe_x_O_y_@CNF cycled in a K half-cell at 50 mA g^−1^ between 0.01 V an 3.00 V. (**g**) Cycle performance and capacity retention of the same K half-cell with MoS_2_@Fe_x_O_y_@CNF compared to those of pristine MoS_2_. Note in (**f**) and (**g**) a severe irreversible capacity loss in the first cycle of MoS_2_@Fe_x_O_y_@CNF, likely due to SEI formation. Reprinted with permission from Ref. [76]. Copyright 2022 Elsevier.

A subsequent work focused on an advanced fabrication via electrospinning and further carbonization at 700 °C under Ar flow of core-shell fiber-type hybrid nanostructures enclosing a dense core of MoS_2_ and Fe_x_O_y_ nanoparticles encapsulated in a smooth and porous outer shell/wall made of carbon [76] (Figure 9–left panel). The corresponding core-shell nanocomposite material with a fibril morphology was referred to as MoS_2_@Fe_x_O_y_@CNF. The role of nanoparticulated MoS_2_ in the preparation of this anodic material was three-fold: (i) It could act as a host for K^+^, (ii) it could improve ion diffusion thanks to its large interlayer spacing and (iii) simultaneously boost the electrochemical performances in presence of carbon nanostructures.

This synthesis route offered an effective full enclosure of the active material inside a smooth carbon nanofiber (CNF) structure that enabled a direct electronic wiring and access of the ions thanks to its intimate contact with the MoS_2_ and Fe_x_O_y_ nanoparticles and its suitable porosity, respectively. Besides, this material design provided a simultaneous aid in containing the volume changes during the electrochemical reactions. The discharge–charge curves of MoS_2_@Fe_x_O_y_@CNF galvanostatically cycled in a K half-cell at 50 mA g^−1^ exhibited an initial discharge capacity around 900 mAh g^−1^ followed by a charge capacity of ≈350 mAh g^−1^ (Figure 9f). Such a severe irreversible capacity in the first cycle was possibly caused by SEI formation. Nevertheless, the MoS_2_@Fe_x_O_y_@CNF electrode could cycle quite well retaining an ultimate capacity of 320 mAh g^−1^ after 100 cycles (Figure 9g) and also withstand cycling at different C-rates from 0.05 C up to 1 C. A synergetic effect of the CNF shell with the MoS_2_ and Fe_x_O_y_ nanoparticles was also demonstrated, since pristine MoS_2_-based electrodes could not attain either high capacities or retain the latter.

**Figure 10:**
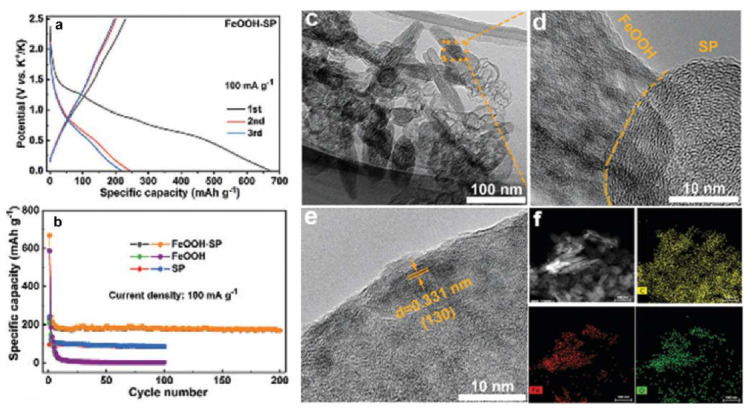
(**a**) Galvanostatic discharge/charge voltage profiles for the first three cycles of the FeOOH-SP composite cycled in a K half-cell at 100 mA g^−1^ between 0.01 V and 2.50 V. (**b**) Cycle performances and capacity retention of the same K half-cell with FeOOH-SP compared to those of the individual β-FeOOH and SP. (**c**) Low-magnification transmission electron microscopy (TEM) image of FeOOH-SP composite and (**d**) magnified detail highlighting respective β-FeOOH and SP morphologies and textures. (**e**) HRTEM showing characteristic lattice fringes for β-FeOOH. (**f**) Elemental mapping showing individual distributions of carbon (yellow), iron (red) and oxygen (green) in the FeOOH-SP composite. Reprinted with permission from Ref. [77]. Copyright 2022 the Royal Society of Chemistry.

A further example of iron oxide-based nanomaterial obtained via a solvothermal process is represented by a β-FeOOH/carbon composite that directly included Super P (SP) as electronically conductive additive in its synthesis procedure to improve the electronic conductivity of the composite and thus facilitate the overall electrochemical reactions [77]. The electrochemical activity of the resulting electrode was enhanced since this type of carbon not only promotes the reactions of FeOOH with K^+^, but can also contribute with possible concurrent K^+^ insertion into its sparse graphitic-like domains. This type of composite with a spindle-like morphology was indicated as FeOOH-SP and its textural and compositional properties are shown in the right hand side of Figure 10.

This study focused on testing a number of electrolyte formulations to improve both storage capacity and cycling stability of the FeOOH-SP electrodes. Through an in situ XRD investigation, it was demonstrated that initial potassiation of FeOOH-SP caused progressive and irreversible amorphization of the β-FeOOH with increasing discharge depths at low potentials vs. K^+^/K. This suggested an irreversible conversion mechanism also displaying a very limited charge recovery in the first cycle (see Figure 10a), probably aggravated by SEI formation. Subsequent galvanostatic cycling at 100 mA g^−1^ between 0.01 and 2.50 vs. K^+^/K resulted in a retention of a moderate capacity of approximately 170 mAh g^−1^ after 200 cycles (Figure 10b). Rate capability tests for FeOOH-SP and long-term cycling at a current density of 1.0 A g^−1^ in K half-cells were also conducted.

**Figure 11:**
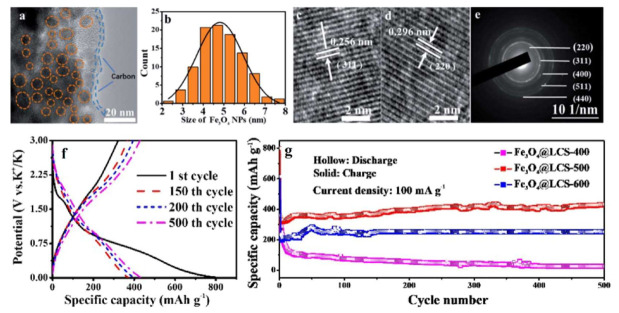
(**a**) High resolution transmission electron microscopy (HRTEM) image of the Fe_3_O_4_@LCS-500 composite highlighting an apparent amorphous carbon layer and a multitude of Fe_3_O_4_ nanodots. (**b**) Size distribution of the small Fe_3_O_4_ nanoparticles, indicating an average size of about 5 nm. HRTEM close-up images (**c**,**d**) showing the crystal fringes and associated crystallographic planes of Fe_3_O_4_. (**e**) Selected area electron diffraction (SAED) pattern with indexed diffraction rings for the series of diffracting crystalline planes. (**f**) Galvanostatic discharge/charge voltage profiles for some selected cycles of Fe_3_O_4_@LCS-500 cycled in a K half-cell at 100 mA g^−1^ between 0.01 V an 3.00 V. (**g**) Cycle performance and capacity retention of the same K half-cell with Fe_3_O_4_@LCS-500 compared to those of Fe_3_O_4_@LCS-400 and Fe_3_O_4_@LCS-600, respectively. Note in (**f**) and (**g**) a prominent irreversible capacity loss in the first cycle of Fe_3_O_4_@LCS-500, possibly due to SEI formation. Images taken and adapted from Ref. [17].

A quite recent investigation lastly reported a synthesis of ultra-small Fe_3_O_4_ nanodots encapsulated in layered carbon nanosheets by employing a favorable coordination interaction between catechol and Fe^3+^ precursors followed by heating at various temperatures (400, 500 and 600 °C) under N_2_ [17]. The resulting nanocomposites were abbreviated as Fe_3_O_4_@LCS. The morphology of the Fe_3_O_4_@LCS-500 nanocomposite, together with its structural properties and characteristic size distribution of its Fe_3_O_4_ nanoparticles are shown in the upper part of Figure 11.

The design of such a hybrid iron oxide/carbon nanocomposite revolved around the possibility of simultaneously improving both electronic and ionic transport properties to support an improved kinetics during the electrochemical reactions, while leveraging on both small size and homogeneous size distribution of the Fe_3_O_4_ nanoparticles to strengthen their overall mechanical behavior, enhancing at the same time their pseudo-capacitive characteristics. The LCS enclosing was also instrumental in alleviating the volume changes induced by cyclic K^+^ uptake and removal. The electrochemical behavior of Fe_3_O_4_@LCS-500 in a K half-cell at 100 mA g^−1^ highlighted a high capacity in the first discharge (≈800 mAh g^−1^), yet an unsatisfactory charge recovery upon subsequent charging (≈320 mAh g^−1^) indicated that irreversible charge losses due to the SEI formation (see Figure 11f,g) could not be prevented also in this case. Interestingly, further cycling resulted not only in stable capacities, but also in a progressive moderate increase of the latter during the remaining 499 cycles with a final value of ≈430 mAh g^−1^. This phenomenon might be due to such a small average size of the Fe_3_O_4_ nanodots (i.e., ≈5 nm), which in principle could yield even smaller (and monodispersed) Fe^0^ nanoparticles in the presence of a full conversion via the K^+^ ions. This, in turn, could activate additional pseudo-capacitive charge storage, as reported also for Fe_3_O_4_@LCS-500 tested in an analogous Li half-cell system, due to the formation of a larger Fe^0^/K_2_O interface and thus additional surface capacitance linked to such ultra-small Fe^0^ nanoparticles, with concomitant anomalous capacity rise through the possible mechanisms [57] described earlier in Section 5.2. The choice of 500 °C for the synthesis of Fe_3_O_4_@LCS-500 appeared also to play a key role in optimizing its cycle performances compared to the other Fe_3_O_4_@LCS nanocomposites obtained at lower and higher temperatures, respectively (Figure 11g). A similar behavior of mild tendency toward capacity increase upon long-term cycling was also reported to occur for the Fe_3_O_4_@LCS-500 electrodes even at higher applied currents (0.5, 1.0 and 2.0 A g^−1^) during 1000 cycles.

### 5.4. Challenges Ahead

From these few studies on composite iron oxide/carbon nanomaterials for potential use as KIB anodes (see also Table 4), it is evident that in all these investigations the theoretical capacities expected from full conversion of these oxides via full potassiation were in practice not reached during the first discharge. This would suggest that, despite a limited size of the Fe_x_O_y_ nanoparticles, the conversion into Fe^0^ and K_2_O through K^+^ is hindered and likely only partial. Conversely, it appeared very challenging to obtain a sufficient reversibility in the first cycle during charging, most likely due to an exacerbated SEI formation and irreversible electrolyte consumption. Even with the most advanced materials design and nano-engineering of these hybrid structures, which effectively prevented the direct contact of the reactive oxide nanostructures with the electrolyte and instead provided a functional encapsulation/protection via different types of carbonaceous materials, the interactions at the K-ion electrolyte/carbon interface appeared not as favorable or manageable as those documented for LIBs. Additionally, the presence of such carbon nanomaterials with an intrinsic large surface area made the SEI issue more pronounced.

Changing the electrolytic species (i.e., Li^+^ for K^+^), even in presence of the same solvent/solvent mixture, has certainly a deep impact on the actual physicochemical properties of the resulting electrolyte [29]. This is reflected unavoidably in the behavior at the liquid–solid interfaces, which are more sensitive to such changes during the electrochemical reactions. Apart from such deviations at the liquid–solid interfaces, the differences in characteristic size, reactivity and diffusivity of Li^+^ and K^+^ clearly play a critical role, as they are directly involved in these solid-state reactions, phase transitions and ultimately generation of the phase boundaries between the Fe^0^ nanoparticles and corresponding alkali oxide. As shown in Table 3 above, an immediate consequence is that, upon progressive reduction of the iron oxide, the space occupied by a newly formed K_2_O phase is much larger compared to that of Li_2_O. Considering also the XRD findings by Sultana et al. [16], it is worth observing that K_2_O—allegedly formed in the initial stages of partial iron oxide conversion—appeared to possess a crystalline nature, or at least its presence could be detected through conventional XRD in the first cycle, unlike Li_2_O under analogous lithiation. This aspect together with a much larger molar volume for K_2_O can reasonably be expected to have an influence on the reaction pathway and extent of conversion. A much larger level of strain due to this extra space taken up by an incipient K_2_O phase could sensibly delay or even halt the whole conversion. This might explain their incomplete reaction in the first cycles and only partially reversible behavior. Similar observations had also been put forward for Na_2_O generation in analogous iron oxide systems in Na half-cells [78,79], thus suggesting that the possibility of achieving a completive mechanism for the conversion of these TMOs cannot discard the size of the guest cation employed in the reactions, as well as the presence of reaction layers that can block the ionic transport [80]. In fact, a full conversion of iron oxide into Fe^0^/Li_2_O requires a significant energy expenditure to form such extended phase boundaries. Nevertheless, by electrochemically driving the reaction it is possible to form an amorphous-like Li_2_O matrix, which can still support Li^+^ ion movement through it, thanks to its nano-sized dimensions and a moderate molar volume, which is incidentally roughly double that of metallic Fe (≈7.09 cm^3^ mol^−1^). The amorphous-like character of Li_2_O could play an additional role in terms of rather isotropic properties of Li^+^ conduction, whereas this might not be the case for a comparatively crystalline K_2_O as analogous K-ion conductor. Creating comparable amounts of phase boundaries for Fe^0^/K_2_O with respect to those of Fe^0^/Li_2_O, while ensuring sufficient transport of K^+^ in K_2_O and e^−^ in Fe^0^, intuitively appears more challenging, especially based on the above arguments (see also Table 3).

These additional challenges for the conversion of iron oxides upon extended potassiation seemingly did not help to improve the cell round-trip efficiency either. No specific data was reported in these early studies on this pertinent aspect; however, a rather consistent voltage hysteresis could be inferred from the galvanostatic voltage profiles of charge and discharge of the K half-cells. Hence, this critical shortcoming should not be neglected in a correct assessment of the actual performances of these materials for their effective use in upcoming KIBs, at least when this round-trip efficiency feature is a key prerequisite for some specific application. In this respect, it should be observed that in the case of LIBs, it is possible to obtain higher round-trip efficiencies in the first cycle for these oxide nanostructures, even in the absence of a carbon coating, by using amorphous nanomaterials, which for example could be produced through electrospray deposition or pyrolysis [64,65] at low or moderate temperatures. Such amorphous iron oxide nanomaterials provided slightly different curvatures and characteristic slopes in their voltage profiles during the first lithiation/de-lithiation cycle and, seemingly, a milder voltage hysteresis compared to their crystalline equivalent. In fact, it was shown that the energy losses of iron oxide during the initial cycle can clearly be reduced by using amorphous iron oxide nanostructures, instead of crystalline ones, since such a crystalline-to-amorphous transition upon initial conversion heavily affects the round-trip efficiency in the first discharge–charge in Li half-cells [65]. This type of irreversible crystalline-to-amorphous transition requires an additional energy expenditure to dismantle the pristine iron oxide crystalline lattice and drive the generation of the Fe nanoparticles surrounded by amorphous-like Li_2_O. Interestingly, the amorphous nature of such iron oxide nanostructures appeared to promote also a progressively rising capacity upon long-term cycling, differently from their crystalline counterpart [65]. Similar considerations are expected to possibly apply to the case of iron oxides cycled with K^+^ ions, too. Potential use of small amorphous iron oxide-based nanoparticles with controlled size and minimal agglomeration, possibly enveloped in a protective surrounding carbon encapsulation would be beneficial to improve not only the initial round-trip efficiency, but also the overall reaction thanks to an improved kinetics and more contained energy expenditure to form Fe^0^/K_2_O with amorphous-like characteristics compared to their crystalline analogues.

Nonetheless, the impact of a rational fabrication of these types of hybrid iron oxide-based/carbon nanostructures proved pivotal in enabling their electrochemical cycling, independently of further amorphous features as possible optimization. In the absence of such an aimed approach with tailored nanomaterials, electrochemical cycling of iron oxides in KIBs would be hardly possible and definitely lead to early cell failure, whereas these initial investigations showed that it is indeed possible to achieve reversible capacities (although sensibly reduced compared to those of analogous LIBs) and even long-term cycling. In light of the above results, relevant improvements have been clearly achieved and this must be acknowledged. The use of dedicated nanostructured routes pushing the limits of the size of the iron oxide nanoparticles, while ensuring their effective local electronic and ionic conduction combined with surrounding voids to accommodate the volume changes, is certainly crucial not only from a practical point of view, but also from a more fundamental perspective. In fact, the observation of an enhanced pseudo-capacitive behavior with progressively rising capacities upon their long-term cycling with both Li^+^ and K^+^ [17] would suggest that a regime in which the charge storage of the reacting nanomaterial becomes mostly interface controlled [81] (e.g., enhanced surface capacitance due to small Fe^0^ nanoparticles) could be reached, despite the extra limitations imposed by the use of K^+^. Moreover, reaching a possible ‘critical size’ and uniform size distribution for the iron oxide nanoparticles, for which the majority of their atoms would be ‘surface atoms’ and thus yielding an enhanced reaction to create smoothly a Fe^0^/Li_2_O or Fe^0^/K_2_O nanocomposite on such (sub)nanoscale lengths, represents another fascinating aspect.

Controlling size, size distribution, dispersion, composition, crystalline/amorphous features, local electronic and ionic wiring of iron oxide-based nanoparticles, which embed sufficient empty space to alleviate their volume changes during the reactions with Li^+^ or K^+^, constitute necessary requisites to achieve a development of these active materials in functional combination with carbon nanostructures to advance their potential use as anodes in LIBs, KIBs and related hybrid electrochemical systems with pseudo-capacitive characteristics.

## 6. Conclusions and Outlook

The application of iron oxide-based materials as potential anodes in both LIBs and possible future KIB technology has carefully been reviewed in this article. The characteristics of natural abundance, cost effectiveness, limited environmental footprint, non-toxicity, safety, high storage capacities and suitability for both large-scale production and eventual recycling of iron oxides makes them certainly attractive for possible uses in advanced rechargeable battery technologies in which the aspect of improved sustainability is taken into account.

However, effective utilization of iron oxides as possible anodes in LIBs and KIBs is evidently not an easy task and this crucial aspect has been clarified for these two specific types of non-aqueous ion chemistries. Intrinsic drawbacks of iron oxides and additional limitations arising from their electrochemical reactions of ion insertion and ultimate conversion have been discussed and outlined.

From a series of relatively recent examples of syntheses of advanced nanostructured iron oxides in combination with carbon nanomaterials in hybrid nanocomposites, it is clear that this type of approach is important to address interesting characteristic issues. Indeed, the cycle performances of such iron oxide-based/carbon nanocomposites appear sensibly improved compared to those of similar nanomaterials that do not employ these types of tailored design and strategies to tackle at once their typical critical points (i.e., volume changes, limited electronic and ionic conductivity, direct contact of the active nanostructures with the electrolyte, particle agglomeration/aggregation). Significant advancements in terms of both nanocomposite designs and possible adaptation of such hybrid nanomaterials to a number of fabrication routes that are compatible and effectively scalable to an industrial production level have definitely been made since their original discovery. Regardless, it should be kept in mind that these types of more sophisticated processes likely involving additional costs and/or presenting low yields could cancel out the inherent advantages of these iron oxide materials, i.e., their low cost and limited environmental footprint.

The perspectives of employing such types of iron oxide-based/carbon nanocomposites in advanced LIB anodes may be quite promising, especially if their specific functionalities could partly deviate from those typically required in conventional negative electrodes and potentially boost the cell rate capabilities via their characteristic pseudo-capacitive charge storage features, instead. This alternative target could help in emphasizing and exploiting this particular aspect of iron oxide conversion mechanisms, instead of relegating this particular type of reconstitution reaction as a ‘pathological stage’ of insertion due to such severe structural changes, amorphization, volume variations and—most importantly—poor round-trip efficiencies. As far as this critical aspect is concerned, it should be stressed once again that this represents an intrinsic disadvantage stemming from the particular nature of the conversion reaction of TMOs, and that, as such, can only be mitigated to some extent, yet not entirely addressed through a sheer materials nano-engineering approach, for example.

Possible use of amorphous iron oxide nanostructures can constitute a feasible way to decrease the energy losses in the first cycle and improve associated round-trip efficiency, thanks to the limited amount of energy required in formatting the oxide into its converted, amorphous-like state (i.e., Fe^0^/Li_2_O nanocomposite), when compared to a typical crystalline-to-amorphous sharp transition during this type of reaction. The amorphous nature of these nanomaterials can help in limiting the energy expenses in generating the reaction products compared to their analogous crystalline form and promoting a more isotropic and effective distribution of the resulting mechanical strain during this reaction. However, this type of structurally amorphous approach would rather constitute only a necessary—yet not sufficient—condition to access decent values of round-trip efficiency in corresponding cells and a trade-off in their ultimate performances is deemed unavoidable, so far.

The energy efficiency figures could incrementally be ameliorated via tailored strategies aiming at improving the conduction of both ions and electrons upon conversion/de-conversion, while protecting the reacting electrode interface in contact with the electrolyte to ensure a smoother charge transfer via optimized ionic and electronic conduction pathways granting a higher level of electrical connectivity throughout the electrode structure. Alternatively, cyclical conversion/de-conversion could purposely be avoided with the aim of accessing only a significantly limited part of the capacity arising from the pseudo-capacitive charge storage in the Fe^0^/Li_2_O nanocomposite at low voltages vs. Li^+^/Li and thus ruling out major structural rearrangements, volume variations, interface reshaping and connected energy losses linked to each full conversion/de-conversion cycle.

An opportunity of bridging the gap between battery and supercapacitor properties could also be offered directly by these iron oxide nanostructures, as they could fit the concepts of: (i) LIBs based on different electrodes with complementary functionalities (i.e., capacity and capacitance) and/or a mix of the latter, or (ii) Li-ion supercapacitors with a metallic lithium anode, provided that its severe interface instabilities, charge losses and safety issues are properly addressed.

Using these types of hybrid Fe oxide/C nanostructures in LIBs is clearly not free from remaining challenges, at least a higher degree of difficulty should be acknowledged for analogous purposes in KIBs. Perhaps this consideration alone might partly justify a current absence of many studies on this specific topic, despite a vast abundance of similar investigations focusing on LIBs. Conversely, the development of non-aqueous KIBs and a quest for suitable anodes represents a technological research area that is flourishing mainly in the last decade. Carbonaceous materials such as graphite are well known to intercalate K^+^ and—as such—have dominated the scene of KIB research on anodic materials. The size differences between Li^+^ and K^+^ constitute even in this well-studied framework of graphite an exemplar case, showing that the dimension of the guest cation for intercalation in the same material does have immediate consequences on the resulting electrochemical behavior, structural stability, interphase properties and ultimate cycling performances.

Iron oxides would be attractive candidates as inexpensive, earth-abundant, environmentally friendly and readily available anode materials aligned with the idea of a possible future cost-effective production of KIBs with complementary purposes to LIBs. Nevertheless, the challenges ahead are multifaceted and an exchange of Li^+^ for K^+^ clearly does not affect positively an already flawed conversion mechanism of these TMOs in terms of remarkable volume expansion/contraction, severe SEI layer formation and interface instabilities yielding limited Coulombic efficiencies together with scarce round-trip efficiencies requiring a large voltage window of operation for capacity delivery. The above considerations and strategies to attempt a modest improvement of this unfavorable energy efficiency figure are certainly valid also in this instance and could help in simultaneously increasing the level of utilization of the corresponding reacted materials, possibly promoting a more extensive potassiation and conversion during their initial cycles, thus supporting slightly better capacities and Coulombic efficiencies as well.

Insights into the limiting factors of iron oxide conversion through uptake of K^+^ and concurrent detailed elucidation of the electrochemical pathways through which the charge storage takes place in these compounds are currently lacking to a rather high extent. In this scenario, possible use of advanced in situ and in operando methodologies can help in gaining a deeper understanding of the details of these complex reactions involving phase transitions and creation of new interphases and interfaces.

## Figures and Tables

**Table 2:** Comparison of some physicochemical properties of Li^+^, Na^+^ and K^+^ ions for possible use in rechargeable non-aqueous batteries.

Guest Cation	Atomic Mass	Density (g cm^−3^)	Ionic Radius (Å)	Melting Point (°C)	E^0^ vs. SHE (V)	E^0^ vs. Li^+^/Li _PC_ (V)	(σ_lim_)_ion_ in PC (S cm^2^ mol^−1^)	References
Li^+^	6.94	0.534	0.76	180.5	−3.04	0	8.3	[68,69,70]
Na^+^	23.00	0.971	1.02	98	−2.71	0.23	9.1	[68,69,70]
K^+^	39.10	0.862	1.38	63	−2.93	−0.09	15.2	[68,69,70]

**Table 3:** Comparison of some physical properties of alkali oxides arising from conversion of iron oxides upon respective lithiation, sodiation and potassiation along with associated theoretical volume changes from their pristine state to their fully reacted and converted states.

Conversion Products	Molar Mass (g mol^−1^)	Density (g cm^−3^)	Molar Volume (cm^3^ mol^−1^)	σ_ion_ at RT (S cm^−1^)	α-Fe_2_O_3_ Volume Change (%)	Fe_3_O_4_ Volume Change (%)	References
Li_2_O	29.88	2.03	14.72	≈1 × 10^−12^	≈92	≈80	[71,72]
Na_2_O	61.98	2.27	27.30	n.a.	≈216	≈193	[73]
K_2_O	94.20	2.30	40.96	n.a.	≈351	≈316	[74]

Note: A molar volume of 7.09 cm^3^ mol^−1^ for α-Fe, 44.53 cm^3^ mol^−1^ for Fe_3_O_4_ and 30.36 cm^3^ mol^−1^ for α-Fe_2_O_3_ were assumed for calculating the volume variations theoretically expected for completive lithiation, sodiation and potassiation, respectively.

**Table 4:** Synoptic comparison of the electrochemical performances obtained in K-ion cells for various types of hybrid nanostructures based iron oxides and carbon materials. These active nanomaterials were tested as potential anodes for KIBs and typically cycled between 0.01 and 3.0 V vs. K^+^/K.

Anode	Electrolyte	Capacity	Stability
Hybrid nano Fe_2_O_3_–Co_3_O_4_/C [16]	0.75 M KPF_6_ in 1:1 (*v*/*v*) mixture of ethylene carbonate (EC)/diethyl carbonate (DEC).	220 mAh g^−1^ at 50 mA g^−1^	50 cycles
Hollow nanospheres of Fe_x_O@NFLG-240 [75]	1 M KFSI in dimethoxyethane (DME).	423 mAh g^−1^ at 50 mA g^−1^; 206 mAh g^−1^ at 2.0 A g^−1^; 140 mAh g^−1^ at 5.0 A g^−1^	100, 1000 and 5000 cycles, respectively.
Core-shell hybrid MoS_2_@Fe_x_O_y_@CNF [76]	1 M KPF_6_ in 1:1 (*v*/*v*) mixture of ethylene carbonate (EC)/dimethyl carbonate (DMC).	320 mAh g^−1^ at 50 mA g^−1^	100 cycles
β-FeOOH-SP carbon composite [77]	1 M KFSI in ethylene carbonate (EC)/diethyl carbonate (DEC) as optimized electrolyte. Other salts and electrolyte formulations were also tested.	≈170 mAh g^−1^ at 100 mA g^−1^; ≈75 mAh g^−1^ at 1.0 A g^−1^	200 cycles and 1000 cycles, respectively.
Ultra-small Fe_3_O_4_@LCS-500 nanocomposite [17]	0.8 M KPF_6_ in 1:1 (*v*/*v*) mixture of ethylene carbonate (EC)/diethyl carbonate (DEC).	430 mAh g^−1^ at 100 mA g^−1^; 141 mAh g^−1^ at 2.0 A g^−1^	500 cycles and 1000 cycles, respectively.

## Data Availability

Not applicable.
